# Muse on Artificial Teeth

**Published:** 1853-04

**Authors:** Samuel D. Muse

**Affiliations:** Sharon, Miss.


[For the Dental Register.

Mr. Editor:—The following is my method of inserting artificial teeth on pivot (which is different in some particulars from that of any other of which I have any knowledge), and has proved very successful in my practice.

The first consideration is a healthy fang and periosteum, and I would always prefer a vital nerve, (I have found that teeth are much more likely to give trouble where the nerve has been lost by suppuration) although the pain attendant on the operation is much greater.

I prepare the fang as is usually practised by most dentists, say excising the crown with file, or saw, and forceps, file up with the stump-file as well as possible, without seriously wounding the periosteum; now drill out the canal with a flat double edged drill, with a projecting point, which I think superior to all others, as the point not only serves to direct the instrument, but also prevents any fragments from being forced out toward the apex of the fang, and thereby involve the loss of the operation; of the drills I have several sizes adapted to the holes in my pivot compress. I now counter drill the end of the root (for which purpose I have several sizes, having four cutting edges, with a center point nearly as large as the canal), about a line, leaving a mere border of dentine to support the crown. I now select and adapt the substitute, get the exact length of pivot, and adapt it to the canal, also to the hole in the tooth, and now being certain that no subsequent hemorrhage or suppuration will ensue, and having prepared some of Evan's Amalgam (Don't say "Quack," for

*We hope that Dr. Todd will watch the further progress of this interesting case, and let us know how many teeth are reproduced. If they have a perfect alveolus, and if the teeth are of the class which should occupy that portion of the jaw, &c. &c.

this is the only purpose for which I use it), with as little mer. cury as possible. I place the pivot home in the root, (and never do I have them so large that I cannot send them home with my thumb and finger). I now place around the pivot enough of the paste to completely fill the countersink. I now apply my crown, and press it up firmly, and all is right; I have a perfect joint, that will exclude effectually both air and water, which is the great desideratum.

In this way I have frequently inserted from one to four teeth in the same mouth without subsequent trouble, but I rarely set more than two on the same day.

Very respectfully, yours,

SAMUEL D. MUSE.

*Sharon, Miss., January 29th,* 1853.

